# Surgery

**Published:** 1880-11

**Authors:** 


### SURGERY.

TREPHINING OF THE SKULL OF A LUNATIC AFTER OLD HEAD-INJURY—COMPLETE RECOVERY.—From the *British Medical Journal* of October 16, we copy this extraordinary and instructive report:

Under the care of Mr. George E. Wherry, surgeon to the hospital, Samuel S., aged thirty-eight, was at work in August, 1878, when a hammer fell six feet on his head. It did not unsense him; but ever after he felt the effects of the blow. At first it was as if he had "a cold in the head." In January, 1879, he was ill in bed for many weeks. After this when he tried to work he was soon obliged to leave off, was attacked by giddiness, by thrills up his back, and by tingling and numbness in his legs. He tried again to work in August, 1879, one year after the injury, but had no idea of what he had to do, and could not fix his mind on anything.

In October, 1879, he came to the hospital complaining of "scrunching" noises in the ears and dragging pains in the vertex, without rest at night; aching pains in both arms and along the insides of the legs, and cold feet. He was admitted into the medical ward, and it was then observed that, of all the symptoms, the most constant and distinct was the "scrunching" feeling in the vertex; and often he placed his fingers over the stellate and adherent cicatrix which marked the hammer-blow. During the last week in 1879 his symptoms were aggravated; he grew irritable and morose, and talked of suicide. The pupil of the left eye was larger, and he had very little sleep.

On January 1, 1880, he made a most determined attempt at suicide by throwing himself over from the staircase at the top of the hospital. His life was saved by the courage of a probationer, Miss Stockburn; but he succeeded in jumping from a lower staircase, and fell fifteen feet, damaging his left ankle. On the following day he was sent to Fullbourn Lunatic Asylum, under the care of Dr. Bacon. The same symptoms continued which have been before described, including the pain in the head in the region of the scar.

He remained in this condition until Dr. Bacon considered that an exploratory operation was to be advised; and accordingly, on March 12, Mr. Wherry removed with the trephine a piece of parietal bone at the seat of injury, and found the dura mater beneath of a deep purple color, but apparently healthy. It bulged, with pulsations, into the wound. The portion of skull removed was three-quarters of an inch in diameter, and had not been fractured. Bleeding vessels were tied with fine hemp thread. Silver wire sutures and carbolized cotton-wool dressings were applied. Ether was given during the operation. The wound healed rapidly and well, and the patient's condition so improved that he went to work in the carpenter's shop attached to the asylum four weeks after the operation. He was discharged from the asylum on June 28, and is now (September 16, 1880) at his regular work as a wood-carver, earning a living for his wife and family.

The operation was undertaken with the hope of removing some source of irritation to the brain which might be found in the skull or dura mater beneath the scar. The history of the case, and the symptoms, although they were more general than local, pointed to the lesion as the cause of his lunacy; and although no source of irritation was discovered, the patient recovered rapidly both his bodily and mental powers after the operation of trephining. The reason for this relief to the brain is not easy to explain, but the facts recorded may be of some interest.

A NOVEL MODE OF TREATMENT OF GONORRHEAL OPHTHALMIA.—Mr. George Critchett, F.R.C.S., reports in the *Lancet* a case of gonorrheal ophthalmia in which he had recourse to a heroic and novel method of treatment. Owing to the extreme acuteness and severity of the symptoms, the difficulty in separating the lids or exposing the cornea, and the impossibility of getting any solution into contact with the conjunctival surface, he had relinquished all hope of saving the sight, and felt justified in adopting any treatment, however severe, that promised a ray of hope. He passed a small silver director under the upper lid as far as the edge of the orbit, against which he kept it pressed, and then with a small sharp-pointed bistoury completely divided the lid perpendicularly as far as the margin of the eyebrow. In order to more completely uncover the cornea he separated the two angles of the divided tarsus and fixed them with fine sutures to the skin of the eyebrow. The cornea looked steamy but not ulcerated, and was buried in chemosed conjunctiva. The immediate effect of this proceeding was to diminish the redness and swelling of the lids and conjunctival membrane, and completely to expose the surface. The subsequent treatment consisted in painting over the entire surface of the conjunctiva three times daily with a solution of nitrate of silver, thirty grains to the ounce, and frequently cleansing and syringing with a solution of alum, ten grains to the ounce. A piece of linen moistened in this solution was kept constantly applied to the eye. This plan was continued, with gradual abatement of the symptoms, for a month; a weaker solution was then substituted. At the end of six weeks from the commencement of the treatment the eye had recovered with a perfectly bright, healthy cornea. At the termination of another fortnight the child was again placed under the influence of an anæsthetic, and the edges of the divided lids were pared and brought together with fine sutures. Good union occurred, the deformity very slight, and the lid perfectly performs its functions. In the early part of the treatment the other eye was kept carefully closed with strapping so as to prevent any risk of inoculation.—*Medical Record.*

CYSTS OF THE MESENTERY. ABLATION BY GASTROTOMY. RECOVERY. By Drs. MILLARD and TILLAUX. (*Bulletin de l' Académie de Médecine.*)

A man of thirty-one years, on the 25th of May, 1880, previously having enjoyed perfect health, was suddenly in the street seized with a pain in the abdomen, so violent that he could not proceed. After a quarter of an hour he succeeded, still "doubled up" with pain, to reach his home. Did not sleep, nor even remain in bed, but passed the whole night rolling on the floor. Several injections were tried without effect, and an obstinate constipation set in and continued from the commencement of the attack of pain.

May 26. No rest was secured, notwithstanding the anodynes which were administered.

May 27. He was taken to the Hospital Lariboisière. A tumor in the right side of abdomen was discovered, and pronounced to be a floating kidney. The pains now came on at intervals of about ten minutes in the twenty-four hours. They were likened by the patient to a fire in the stomach and bowels.

June 11, he placed himself under care of Dr. M. Millard. The periodic pain was still present, he could not sleep for more than two hours at a time, and even then seated in bed and his head bent on his knees. The horizontal position immediately produced suffocation.

The tumor in the abdomen, on the right side of the umbilicus, was about the size of the head of a fœtus. The patient repeatedly affirmed that he had never noticed it before the attack of pain above referred to. It was round, smooth, very movable and not tender on pressure. After several days Dr. Millard, from the sudden appearance of the difficulty, the abdominal pain and persistent constipation, diagnosed chronic invagination of the intestine.

The treatment adopted was the use of the constant current, massage of the abdominal region, enemas of various kinds. No benefit followed; the pains were just as severe; the patient could eat nothing, and for forty days had not had a passage of the bowels without the aid of an injection.

He was then transferred to the service of Dr. Tillaux, and although he got a little better for a few days, the same pains recurred, and the patient demanded some operation for his relief.

July 3. Gastrotomy was performed by Dr. Tillaux, in the presence of Drs. Millard, Féréol and Peyrol, the object being to disinvaginate, if possible, if not, to cut out and apply sutures.

The abdomen being well opened we discovered, instead of invagination, a tumor of the mesentery.

The notes taken by the interne are as follows:

Anæsthesia by chloroform; the abdominal walls shaven and washed with 20 per cent. of carbolic acid. The incision, in median line, about fifteen centimeters; not much hæmorrhage. The peritoneum taken up with a forceps and opened. Abdominal cavity explored with the hand. Incision, increased with a scissors five or six centimeters, allowed a round, smooth tumor about the size of the head of a fœtus to escape. It was situated in the mesentery, and not adherent to the intestines. It was red and vascular on the surface.

Small trocar inserted, but no discharge until the trocar was withdrawn, when a jet of yellowish, creamy fluid appeared. The intestines were protected with warm napkins and the tumor incised, and the whole of the creamy fluid removed. The pedicle was then ligatured with seven pieces of catgut, the walls cut off with scissors and returned to the abdomen. The blood was removed from the abdomen with a sponge, and sutures embracing the entire thickness of the abdominal walls were inserted to close the wounds. Lister's dressing applied.

During the day of operation two hypodermic injections of morphia. No further pain, no vomiting. Slept several hours at a time, and smoked his pipe.

July 4. Skin moist, normal facial expression, pulse 120; temperature, morning, 38.2; evening, 38.8.

The remaining part of the notes do not differ from the general reports on gastrotomy.

August 1, the patient was well, and on the 17th presented himself before the Académie de Médecine when the report was read.

RARE FORM OF DIFFUSE PHLEGMON.—(M. Broca, *Journal de Médecine*, p. 351. Aug., 1880).

A man of forty entered the service at Necker Hospital with a diffuse phlegmon of the arm of a peculiar form, as well because of its etiology as its special aspect. This man had received no blow, wound or injury of any kind, but several days previous had felt a pretty sharp pain in the back of his left hand. The affection seemed to have begun deep in the tissues and spontaneously. The only cause to which it could be imputed was an excess of work which consisted in lifting pieces of metal. Now an excess of this kind may readily bring on inflammation of that sort of serous sac, which covers the extensor tendons at their lower extremities; this sac presents no regularity; it is rather loose cellular tissue than a true serous membrane, but one may nevertheless compare what has happened to what is produced in the majority of phlegmons of the arm. In this case indeed, the inflammation is nearly always due to a solution of continuity of the skin complicated with angioleucitis, or to a contusion and inflammation of the serous sac of the olecranon; now here it is still a serous membrane of a peculiar nature, which has been the point of departure of the trouble, and under the influence of an injury less frequent than the others. There has been from the beginning, redness and swelling of the back of the hand; this extended to the forearm and has made a very threatening progress toward the arm. In this part the swelling had assumed as regards its form a very remarkable character; the swelling was circular and had extended upward around the arm like a muff, limited brusquely as by a ligature. This character of muff-like swelling which has been well described by Chassaignac, has been especially observed in the diffuse periostitic phlegmons of limbs with a single bone. It may, however, show itself under other conditions, and is recognized by the fact that immediately above it the form of the limb is not altered. The skin is not red, and the œdema presents the aspect of that which is encountered as the result of profound compressions.

In the patient thus attacked, the general condition is grave, and there are, moreover, formidable local accidents to be feared. If the disease is to be left to itself, at the end of 24 hours, the muff would have reached the shoulder and intervention would perhaps be too late. Gangrene is imminent, and not only the skin would be sphacelated, but also the entire member would be in such a state as to require disarticulation of the shoulder. To remedy such a situation there are no other means than incisions. A single one is sufficient, as in the present case, when it is seen to allow the ready escape of the fluids which infiltrate the parts, and diminishes the tension. If not, several incisions should be made. The incisions always bleed a good deal because the vessels, fixed by adhesions, do not contract. Therefore one should carefully watch the escape of blood which speedily becomes excessive and might compromise the life of the patient.

DIASTASIS OF THE FOOT.—(M. Broca, *Journal de Médecine*, p. 352, Aug., 1880.)

The peculiar anatomical conditions of the foot are such that injuries may produce lesions of a somewhat special nature. M. Broca had in his service a man who had been hurt in attempting to stop a runaway horse. At first the swelling did not allow detection of anything except mobility without crepitation, but at the end of some days, he was able to determine the lesion in a more precise manner, and found the same symptom still more clearly marked; nearly always abnormal mobility without crepitation indicates the existence of an articular lesion; still the symptom may be observed in fractures of the metatarsals, and is precisely what renders the differential diagnosis difficult. In this case it was easy to see that the mobility existed between the first cuneiform and the first metatarsal; to find the exact seat of this articulation, there is one point important to remember; it is that it is found precisely at the middle of the internal border of the foot. This point, indicated by Blandin, may be very useful in operative medicine, for the swelling may easily cause others to disappear. In this case then it was exactly at this point that the mobility was located; there was a displacement vertically causing an elevation of about half a centimeter (1-5 inch).

To explain this displacement we must admit, as a consequence, the rupture of the ligaments, and it is this constitutes diastasis, properly speaking, a more considerable lesion than simple sprain, since there is displacement, and less than dislocation since the displacement is not maintained. Immobilization and repose are the only means to be employed in such lesions, which ordinarily heal quicker than a fracture in the same region. That is the principal interest there is in establishing a precise diagnosis.

